# TNF-α synergises with IFN-γ to induce caspase-8-JAK1/2-STAT1-dependent death of intestinal epithelial cells

**DOI:** 10.1038/s41419-021-04151-3

**Published:** 2021-09-23

**Authors:** Jerzy A. Woznicki, Nisha Saini, Peter Flood, Subhasree Rajaram, Ciaran M. Lee, Panagiota Stamou, Agnieszka Skowyra, Milan Bustamante-Garrido, Karine Regazzoni, Nyree Crawford, Simon S. McDade, Daniel B. Longley, Pedro Aza-Blanc, Fergus Shanahan, Syed A. Zulquernain, Jane McCarthy, Silvia Melgar, Bradford L. McRae, Ken Nally

**Affiliations:** 1 APC Microbiome Ireland, University College Cork, Cork, Ireland; 2 Centre for Cancer Research and Cell Biology, Queen’s University Belfast, Belfast, UK; 3 Sanford Burnham Prebys Medical Discovery Institute, La Jolla, CA 92037 USA; 4 Department of Medicine, University College Cork, Cork, Ireland; 5 Department of Gastroenterology, Mercy University Hospital, Cork, Ireland; 6 Immunology Discovery, Abbvie Bioresearch Center, Worcester, MA 01605 USA; 7 School of Biochemistry and Cell Biology, University College Cork, Cork, Ireland

**Keywords:** Necroptosis, Cell death and immune response, Interferons, Tumour-necrosis factors, Crohn's disease

## Abstract

Rewiring of host cytokine networks is a key feature of inflammatory bowel diseases (IBD) such as Crohn’s disease (CD). Th1-type cytokines—IFN-γ and TNF-α—occupy critical nodes within these networks and both are associated with disruption of gut epithelial barrier function. This may be due to their ability to synergistically trigger the death of intestinal epithelial cells (IECs) via largely unknown mechanisms. In this study, through unbiased kinome RNAi and drug repurposing screens we identified JAK1/2 kinases as the principal and nonredundant drivers of the synergistic killing of human IECs by IFN-γ/TNF-α. Sensitivity to IFN-γ/TNF-α-mediated synergistic IEC death was retained in primary patient-derived intestinal organoids. Dependence on JAK1/2 was confirmed using genetic loss-of-function studies and JAK inhibitors (JAKinibs). Despite the presence of biochemical features consistent with canonical TNFR1-mediated apoptosis and necroptosis, IFN-γ/TNF-α-induced IEC death was independent of RIPK1/3, ZBP1, MLKL or caspase activity. Instead, it involved sustained activation of JAK1/2-STAT1 signalling, which required a nonenzymatic scaffold function of caspase-8 (CASP8). Further modelling in gut mucosal biopsies revealed an intercorrelated induction of the lethal CASP8-JAK1/2-STAT1 module during ex vivo stimulation of T cells. Functional studies in CD-derived organoids using inhibitors of apoptosis, necroptosis and JAKinibs confirmed the causative role of JAK1/2-STAT1 in cytokine-induced death of primary IECs. Collectively, we demonstrate that TNF-α synergises with IFN-γ to kill IECs via the CASP8-JAK1/2-STAT1 module independently of canonical TNFR1 and cell death signalling. This non-canonical cell death pathway may underpin immunopathology driven by IFN-γ/TNF-α in diverse autoinflammatory diseases such as IBD, and its inhibition may contribute to the therapeutic efficacy of anti-TNFs and JAKinibs.

## Introduction

Excessive death of intestinal epithelial cells (IECs) is associated with severe gut immunopathology in inflammatory bowel diseases (IBD), including Crohn’s disease (CD) [1]. On a molecular level, IBD is associated with rewiring of host cytokine networks, including a shift towards a mixed Th1-type cytokine profile (such as IFN-γ and TNF-α) particularly in CD [2]. TNF-α, a clinically validated therapeutic target in CD [3], is predominantly an inflammatory cytokine that signals through NF-κB and MAPK pathways to regulate inflammatory and survival gene expression. While most cell types are resistant to its direct cytotoxic effects, TNF-α can trigger context-dependent cell death downstream of TNF receptor 1 (TNFR1) in cells sensitised through largely non-physiological perturbations [4]. Under these conditions, TNF-α can induce apoptosis via TRADD-dependent complex IIa or RIPK1/FADD-dependent complex IIb (ripoptosome), or it can promote RIPK3/MLKL-dependent necroptosis [5]. The pathophysiological function of these death complexes in the gut was underscored by studies, in which IEC-specific depletion of NF-κB-related genes [6–8], caspase-8 [9], FADD [10], cFLIP [11] or RIPK1 [12, 13] sensitised mice to TNF-α-mediated intestinal inflammation and cell death. In humans, increased IEC apoptosis was observed in the inflamed gut of patients with CD [14], and this aberrant response was normalised by anti-TNFs [15]. TNFR1 inhibition failed, however, to fully restore homeostasis in the above models of intestinal inflammation [7, 8, 10–13], and anti-TNFs are indeed often ineffective in patients with CD [16, 17]. This highlights a critical role for other drivers of gut immunopathology that are either independent of or redundant with TNF-α. Based on its synergistic lethal interaction with TNF-α in IECs [18–21], IFN-γ may function as such a mediator [17].

In all cell lineages, the binding of IFN-γ to its receptor complex leads to the activation of tyrosine kinases JAK1 and JAK2, which in turn facilitates recruitment and Y701 phosphorylation (and thus activation) of STAT1—a transcription factor central to regulating cellular responses to IFN-γ [22]. Direct cytotoxicity of IFN-γ in the inflamed gut extends to both IECs (including goblet or Paneth cells) and intestinal stem cells [20, 23–26]. Although these deleterious effects are linked to induction of apoptosis, IFN-γ—similar to TNF-α—can also promote RIPK1/3-dependent necroptosis [27–29]. Of note, elevated levels of IFN-γ-secreting cells [30–32] and increased STAT1 activation [33] are often found in CD lesions, while genes encoding members of the IFN-γ pathway overlap IBD-general (*IFNG*, *JAK2* and *STAT1*) or CD-specific (*IFNGR2*) susceptibility loci [34].

While IFN-γ can promote intestinal damage in its own right, IEC injury is often amplified through its synergistic interaction with TNF-α. This interplay was initially associated with apoptosis-independent disruption of epithelial barriers [35]. However, it is now clear that IFN-γ/TNF-α can also cooperate to trigger synergistic death of IECs [18–20], a phenotype retained in primary human colonic organoids [21]. Unlike non-cytotoxic barrier disruption, the exact mechanisms underpinning this lethal response, while linked to feedback inhibition of Wnt/β-catenin signalling [20], remain elusive. Nonetheless, studies in hepatocytes [36], β cells [37] and more recently IECs [21] position STAT1 as one of the key effectors of IFN-γ/TNF-α-induced synergistic killing. Inadequate control of STAT1-mediated transcription in IECs was, in fact, sufficient to sensitise mice to TNF-α-driven intestinal damage, which was attenuated in STAT1^−/−^ animals [38]. While inhibition of either Th1-type cytokine is an effective monotherapy in experimental IBD [39], preclinical data also support the role of IFN-γ/TNF-α cooperativity (if not synergy) in IBD immunopathology [40]. Recently, expansion of IFN-γ-producing TNFR2^+^IL23R^+^ T cells was linked to resistance to anti-TNFs in CD [17], further underscoring the disease relevance of IFN-γ/TNF-α interaction.

We, therefore, reasoned that the under-explored mechanisms required for synergistic killing of IECs by IFN-γ/TNF-α may offer insights into actionable effectors that drive immunopathology of the epithelium in CD. To systematically identify them, we performed kinome RNAi and drug repurposing screens that discovered JAK1/2 as the key nonredundant drivers of IFN-γ/TNF-α-induced synergistic lethality in human IECs. A combination of genetic and pharmacological perturbations in human colonic cell lines and primary organoids, coupled with ex vivo modelling of Th1-type immune responses in mucosal biopsies revealed that IFN-γ/TNF-α synergise to dose-dependently kill IECs via the CASP8-JAK1/2-STAT1 module. This non-canonical TNFR1-mediated cell death pathway is independent of RIPK1/3, ZBP1, MLKL or caspase activity, and can be blocked by clinically relevant JAKinibs under development for CD.

## Results

### IFN-γ and TNF-α synergise to kill IECs in a dose-dependent manner

**Fig. 1:**
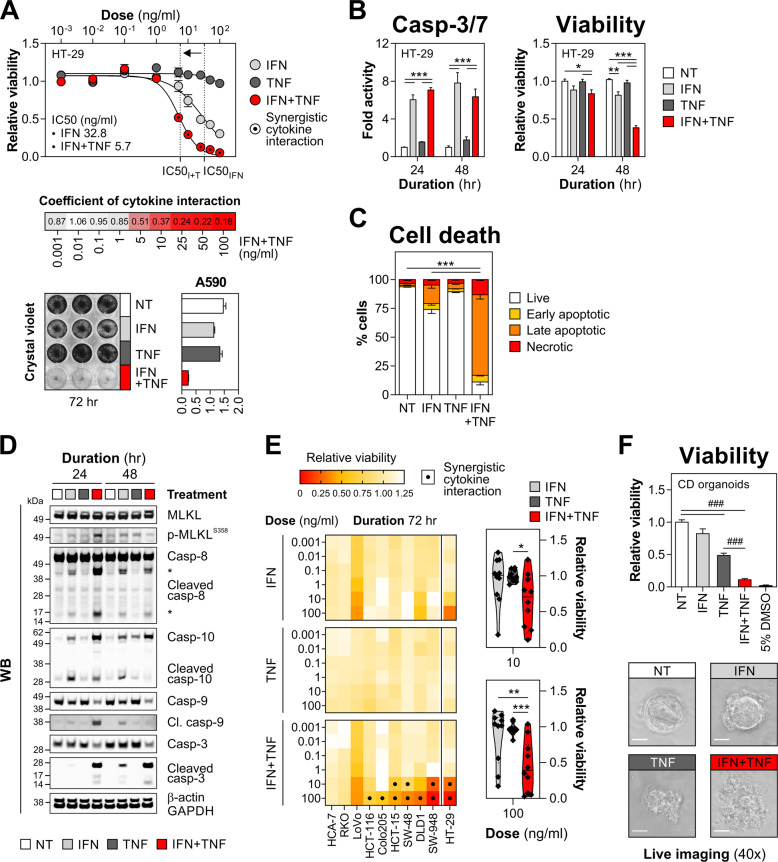
IFN-γ synergises with TNF-α to kill IECs in a dose-dependent manner. **A** HT-29 cells were treated with increasing concentrations of IFN-γ and/or TNF-α for 72 h. Relative viability, including absolute IC_50_ values (top) and a heat map of viability-based coefficients of cytokine interaction (middle). Crystal violet staining and its absorbance quantification (A590 nm) at 10 ng/ml cytokine dose (bottom). **B**–**D** HT-29 cells were treated with IFN-γ and/or TNF-α (10 ng/ml each) for the indicated times. **B** Caspase-3/7 activity and relative viability of HT-29 cells measured at 24 and 48 h. **C** HT-29 cells were stained for active caspase-3 and fixable viability dye FVS660 at 72 h, analysed by flow cytometry and percentages of live/dying cells were quantified. Data were mean ± SEM of *n* = 2 independent experiments. **D** Western blots showing levels of MLKL (total and S358-phosphorylated) and CASP3/8/9/10 (total and cleaved) in HT-29 cells treated as indicated for 24 and 48 h. **E** Colonic cell lines were treated with increasing concentrations of IFN-γ and/or TNF-α for 72 h. Heat maps showing relative viability in each line (left) and a combined viability score across the cell line panel (including HT-29 data, extracted from section (A)) for the indicated cytokine doses (right). **F** Primary human colonic organoids derived from Crohn’s disease (CD) colonic biopsies (*n* = 3 lines) were treated with IFN-γ and/or TNF-α (10 ng/ml each) or 5% DMSO for 72 h. Relative viability (top) and representative live images of a select organoid line (bottom). Unless specified otherwise, data were mean ± SEM of *n* = 3 independent experiments. *p < 0.05, **p < 0.01 and ***p < 0.001 (two-way ANOVA with Tukey’s multiple comparisons test), ^###^*p* < 0.001 (one-way ANOVA with Tukey’s multiple comparisons). NT non-treated.

Modelling of cytokine-induced intestinal damage revealed that TNF-α was well-tolerated by HT-29 cells, while the cytotoxic activity of IFN-γ was limited to high doses (IC_50_ 32.8 ng/ml, Fig. 1A). In contrast, dual treatment with IFN-γ/TNF-α triggered robust cell killing as evidenced by an IC_50_ shift to 5.7 ng/ml, and only residual viability (<10% of a non-treated control) was detected following stimulation with 25–100 ng/ml of both cytokines (Fig. 1A). This dose-dependent lethal response driven by synergistic interaction of IFN-γ/TNF-α, resulted in a complete loss of cellular adherence (Fig. 1A) and increased caspase-3/7 activity (Fig. 1B). In line with its limited killing capacity, IFN-γ triggered a modest accumulation of early/late apoptotic and necrotic cells (Figs. 1C and S1). Populations of late apoptotic and necrotic cells, however, significantly expanded in dually treated HT-29 cultures (Figs. 1C and S1), which displayed biochemical features of TNF-α-mediated apoptosis (cleavage of caspase-8/10/9/3) and necroptosis (MLKL^S358^ phosphorylation) (Fig. 1D) [41]. Considering widespread cell line heterogeneity, including outlier kinase expression that can confer resistance to cell death triggers [42], we profiled sensitivity to IFN-γ/TNF-α in a panel of nine additional colonic cell lines. While some lines were resistant to dual cytokine treatment (HCA-7 and RKO) or were primarily IFN-γ-sensitive (LoVo), the synergistic killing effect of IFN-γ/TNF-α was retained in the majority of tested cell lines (Fig. 1E). Likewise, primary patient-derived colonic organoids, though partially sensitive to TNF-α, proved equally susceptible to IFN-γ/TNF-α (Fig. 1F). Together, IFN-γ/TNF-α synergised to dose-dependently kill IECs, and this damage response was functionally retained in human colonic cell lines and primary organoids.

### JAK1 and JAK2 drive synergistic killing of IECs by IFN-γ/TNF-α

**Fig. 2:**
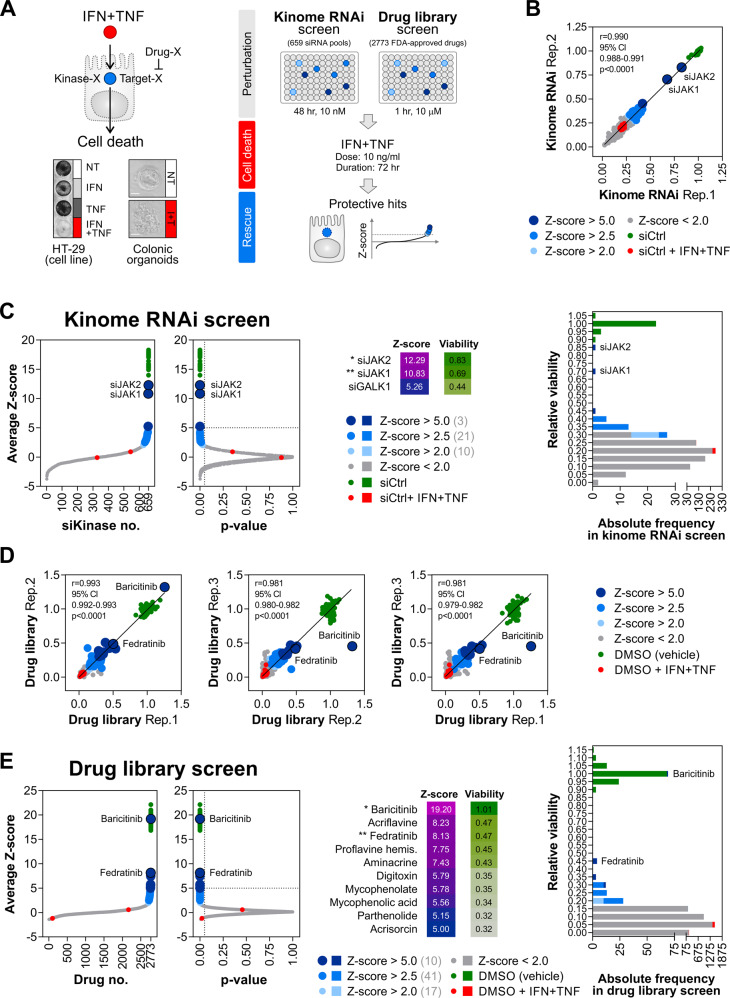
Functional screening identifies JAK1/2 as key drivers of IEC death induced by IFN-γ/TNF-α. **A** Schematic workflow of the kinome RNAi and drug library rescue screens performed in HT-29 cells against a fixed cell death trigger: IFN-γ/TNF-α, 72 h, 10 ng/ml each. **B** Interreplicate reproducibility in the kinome RNAi screen. **C** *Z*-score distribution and its associated *p* values across the kinome RNAi library (left). Average relative viability for candidate genes with *Z*-score >5.0 (middle). Frequency distribution of average relative viability across the kinome RNAi library (right). **D** Interreplicate reproducibility in the drug library screen. **E** *Z*-score distribution and its associated *p* values across the drug library (left). Average relative viability for candidate drugs with *Z*-score >5.0 (middle). Frequency distribution of average relative viability across the drug library (right).

Cell death signalling initiated by either cytokine relies on context-dependent kinase utilisation (RIPK1/3 by TNF-α [41] and JAK1/2 by IFN-γ [22] in particular). Considering this, we reasoned that kinases are likely to form nonredundant signalling hubs required for synergistic killing of IECs by IFN-γ/TNF-α. To test this hypothesis, we completed a genetic rescue screen in HT-29 cells using a siRNA library targeting 659 human genes annotated as 'kinases' (Fig. 2A) against the synergistic cell death trigger: IFN-γ/TNF-α (72 h, 10 ng/ml each). Performed in duplicate (Fig. 2B), the kinome RNAi screen identified 34 protective candidate genes (*Z*-score >2.0 and *p* value < 0.05, Fig. 2C and Table S1). To isolate high-confidence hits, we required cellular viability to be restored to at least 50% of the baseline control. This exercise identified only 2 kinases—JAK1 and JAK2—as positive regulators of IFN-γ/TNF-α-induced killing (Fig. 2C). Thus, the synergistic cell death signal was either fit to bypass signalling by other kinases in the context of their individual depletion, or the underrepresentation of otherwise high-confidence hits arose due to false-negative events. Given this caveat and to improve on the translational potential of our study, we complemented the kinase-focused genetic approach with an orthogonal drug repurposing screen (Fig. 2A) against the same cell death trigger (IFN-γ/TNF-α, 72 h, 10 ng/ml each). Using a curated library of 2773 FDA-approved drugs, we performed a triplicate screen in HT-29 cells (Fig. 2D) and identified 68 protective candidate compounds (*Z*-score >2.0 and *p* value < 0.05, Fig. 2E and Table S2). After analogous high-confidence filtering, only one compound—a dual JAK1/2 inhibitor baricitinib—conferred satisfactory resistance to IFN-γ/TNF-α (Fig. 2E). Together, genetic and pharmacological screens for unbiased identification of drivers of IFN-γ/TNF-α-induced IEC death converged on IFN-γ-regulated kinases JAK1/2.

**Fig. 3:**
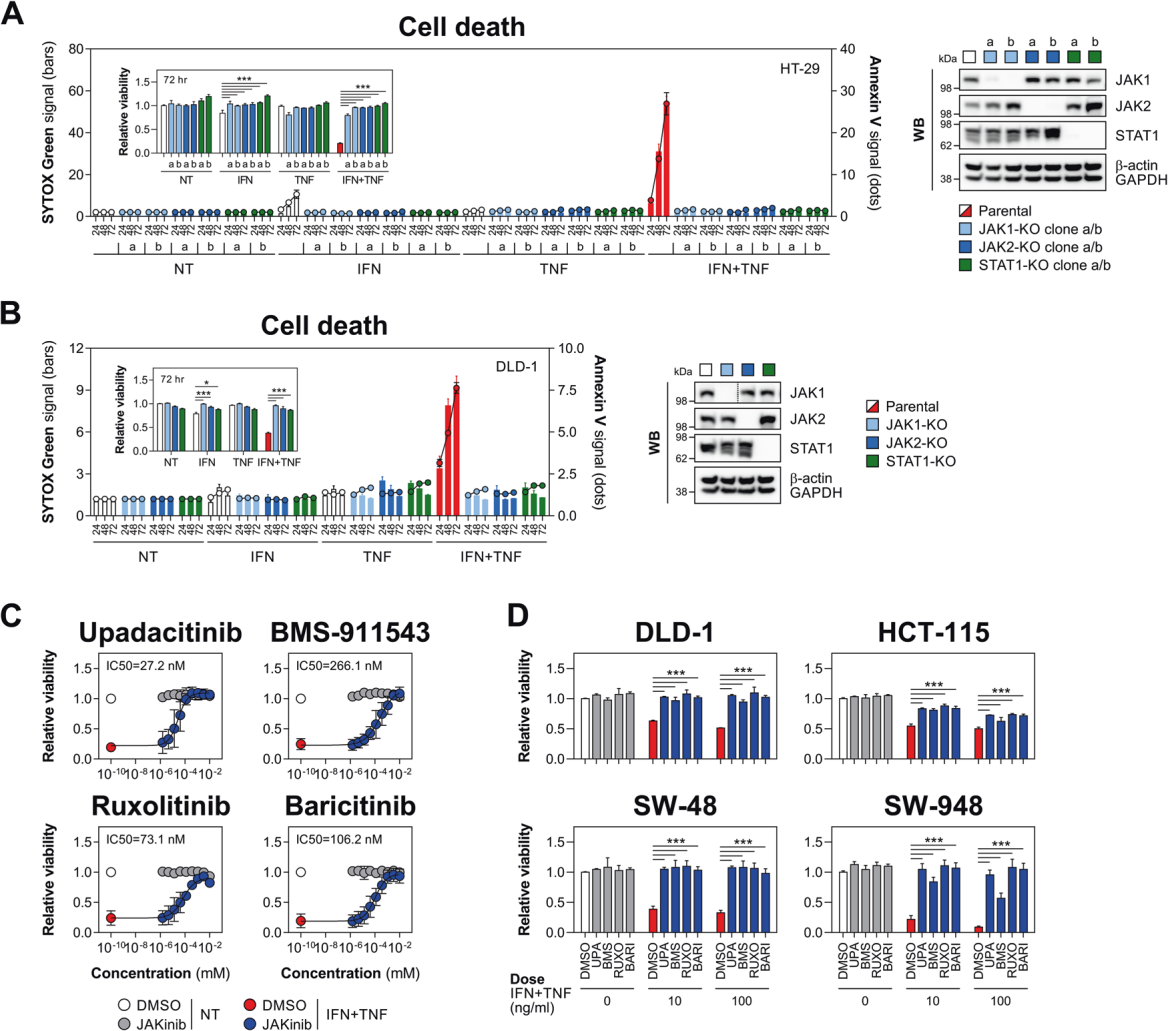
JAK1/2 inhibition protects IECs from IFN-γ/TNF-α. **A** Parental, JAK1, JAK2 or STAT1 knockout HT-29 cells were treated with IFN-γ and/or TNF-α (10 ng/ml each) for up to 72 h. SYTOX Green/Annexin V signals were recorded at indicated times, and relative viability was measured at 72 h (inner graph). Two knockout clones per each target (labelled as 'a' and 'b') were generated. Western blot validation of target knockout (right). **B** Parental, JAK1, JAK2 or STAT1 knockout DLD-1 cells were treated with IFN-γ and/or TNF-α (100 ng/ml each) for up to 72 h. SYTOX Green/Annexin V signals were recorded at indicated times, and relative viability was measured at 72 h (inner graph). Western blot validation of target knockout (right). **C** Relative viability of HT-29 cells pretreated for 1 h with vehicle (DMSO), JAK1-selective compound (upadacitinib), JAK2-selective compound (BMS-911543) or dual JAK1/2 inhibitors (ruxolitinib or baricitinib) starting at 10 μM with threefold dilutions down to 1.52 nM, followed by IFN-γ/TNF-α (10 ng/ml each) for 72 h. Data were mean ± SD of *n* = 3 independent experiments. **D** Relative viability of DLD-1, HCT-115, SW-48 and SW-948 cells pretreated for 1 h with DMSO or the indicated JAK inhibitors (1 μM each), followed by IFN-γ/TNF-α (10 or 100 ng/ml each) for 72 h. Unless specified otherwise, data were mean ± SEM of *n* = 3 independent experiments. *p < 0.05 and ***p < 0.001 (two-way ANOVA with Tukey’s multiple comparisons test). NT non-treated, KO knockout, JAKinib JAK inhibitor, UPA upadacitinib, BMS BMS-911543, RUXO ruxolitinib, BARI baricitinib.

To validate this functional dependence, we established CRISPR/Cas9 knockout clones for JAK1/2 and STAT1 in HT-29 and DLD-1 cell lines. As expected, depletion of either kinase or STAT1 rescued both lines from IFN-γ/TNF-α, while having no impact on baseline survival or sensitivity to either cytokine alone (Fig. 3A, B). We confirmed that the observed protective effects were due to impaired JAK1/2 or STAT1 function rather than casual clonal differences by targeting all three genes with siRNA (Fig. S2A). Because our goal was to identify actionable drivers of IFN-γ/TNF-α-induced killing, we next validated JAK1/2 dependence using clinically relevant JAKinibs. The inhibitor panel included JAK1-selective compounds (upadacitinib and filgotinib), JAK2-selective compounds (BMS-911543 and fedratinib) and dual JAK1/2 inhibitors (ruxolitinib and baricitinib), and was tested against a fixed lethal dose of IFN-γ/TNF-α in HT-29 cells. The concentration-response analysis revealed that upadacitinib (IC_50_ 27.2 nM), BMS-911543 (IC_50_ 266.1 nM), ruxolitinib (IC_50_ 73.1 nM) and baricitinib (IC_50_ 106.2 nM) prevented cytokine-induced killing, with upadacitinib being the most potent JAKinib (Fig. 3C). By contrast, filgotinib marginally restored cell viability, while fedratinib showed a narrow activity window (41.2-1111.1 nM, IC_50_ 182.4 nM) (Fig. S2B). These results were consistent with the effect of JAKinibs on cytokine-induced STAT1 activation (Fig. S2C). To increase the stringency of this approach, we extended validation of JAKinibs to the colonic cell lines synergistically sensitive to IFN-γ/TNF-α (Fig. 1E). Similar to HT-29 cells, upadacitinib, BMS-911543, ruxolitinib and baricitinib protected those additional lines from lethal cytokine doses (Fig. 3D). Together, both genetic and pharmacological validation confirmed JAK1/2 as the principal and nonredundant drivers of synergistic IEC killing by IFN-γ/TNF-α.

### IFN-γ and TNF-α kill IECs via non-canonical TNFR1-mediated signalling

**Fig. 4:**
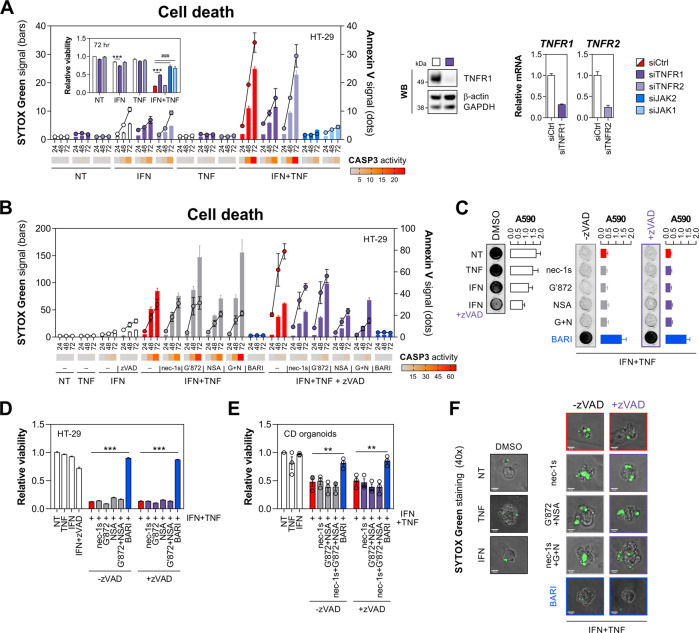
IFN-γ and TNF-α kill IECs through non-canonical TNFR1 signalling. **A** HT-29 cells were transfected with a nontargeting siRNA (siCtrl), siTNFR1, siTNFR2 or positive control siRNA’s (siJAK1, siJAK2), followed by IFN-γ/TNF-α (10 ng/ml each). SYTOX Green/Annexin V signals and caspase-3 activity (heatmap) were recorded at indicated times, and relative viability was measured at 72 h (inner graph). Western blot and RT-qPCR validation of TNFR1/2 knockdown (right). **B**–**D** HT-29 cells were pretreated for 1 h with vehicle (DMSO), RIPK1 inhibitor (necrostatin-1s, 10 μM), RIPK3 inhibitor (GSK′872, 10 μM), MLKL inhibitor (NSA, 1 μM), dual JAK1/2 inhibitor (baricitinib, 10 μM) alone or in combination with a pan-caspase inhibitor (zVAD, 20 μM), followed by IFN-γ/TNF-α (10 ng/ml each). **B** SYTOX Green/Annexin V signals and caspase-3 activity (heatmap) were recorded at indicated times. **C** Crystal violet staining and its absorbance quantification (A590 nm) at 72 h. **D** Relative viability measured at 72 h. **E**, **F** Primary human colonic organoids derived from CD colonic biopsies (*n* = 3 lines) were pretreated for 30 min with the indicated inhibitors (concentrations as in section B), followed by IFN-γ/TNF-α (10 ng/ml each). **E** Relative viability and **F** SYTOX Green staining were assayed at 8 h after cytokine treatment. Data were mean ± SEM of *n* = 3 independent experiments. **p < 0.01 and ***p < 0.001 (two-way ANOVA with Tukey’s multiple comparisons test), ^###^*p* < 0.001 (unpaired *t*-test). NT non-treated, nec-1s necrostatin-1s, G′872 GSK′872, G + N GSK′872 + NSA, BARI baricitinib.

Considering the central role of RIPK1/3 during TNFR1-mediated canonical cell death signalling [41], we found it surprising neither kinase was identified as a protective hit in the kinome RNAi screen. Having confirmed that IFN-γ/TNF-α-induced killing depended on TNFR1 (Fig. 4A), we targeted key checkpoints of TNF-α-regulated cell death to gain further mechanistic insight into cell fate decisions. Initially, we found that a pan-caspase inhibitor zVAD when combined with either necrostatin-1s (RIPK1 inhibitor) or NSA (MLKL inhibitor) partially reduced IFN-γ/TNF-α-driven SYTOX Green cell death signal (Fig. 4B). However, these or indeed any other tested perturbations failed to offer robust protection from IFN-γ/TNF-α (Fig. 4C, D), thus excluding the role for canonical TNFR1 signalling through complex IIb (ripoptosome) or necrosome. Likewise, zVAD alone had no impact on sensitivity to IFN-γ/TNF-α (Fig. 4C, D), suggesting that pro-apoptotic complex IIa was also not essential, and that—unlike other systems [43]—caspase inhibition did not result in rebound necroptosis. Caspase inhibition did, however, further increase the IFN-γ/TNF-α-driven Annexin V signal (Fig. 4B), which is consistent with previous reports on enhanced exposure of phosphatidylserine by IFN-γ also in the presence of zVAD [44]. In line with no protective effect of apoptosis/necroptosis inhibitors, we found that depletion of RIPK1, RIPK3 or ZBP1 with siRNA also failed to rescue HT-29 cells from IFN-γ/TNF-α (Fig. S3A). Instead, classical sensitisers to TNF-α-induced killing – cycloheximide (translational inhibitor), BV6 (SMAC mimetic) or IKK-16 (IKK inhibitor)—all predisposed HT-29 cells to canonical TNF-α-mediated cell death, which was not rescued by JAK1/2 inhibition (Fig. S3B). To verify our mechanistic findings in a primary model of IEC, we found that IFN-γ/TNF-α also killed CD-derived colonic organoids even when apoptotic/necroptotic signalling was co-inhibited (Fig. 4E, F).

**Fig. 5:**
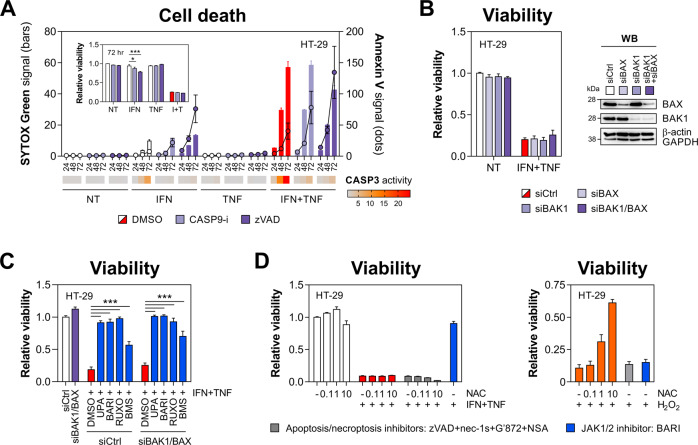
IFN-γ and TNF-α kill IECs independently of mitochondrial apoptosis regulators. **A** HT-29 cells were pretreated for 1 h with DMSO, caspase-9 inhibitor (Z-LEHD, 10 μM) or zVAD (20 μM), followed by IFN-γ/TNF-α (10 ng/ml each). SYTOX Green/Annexin V signals and caspase-3 activity (heatmap) were recorded at indicated times, and relative viability was measured at 72 h (inner graph). **B** Relative viability of HT-29 cells transfected with siCtrl, siBAK1 or siBAX, followed by IFN-γ/TNF-α (10 ng/ml each) for 72 h (left). Western blot validation of BAK1/BAX knockdown (right). **C** Relative viability of HT-29 cells transfected with siCtrl or siBAK1/BAX, subsequently pretreated for 1 h with DMSO, JAK1-selective compound (upadacitinib), JAK2-selective compound (BMS-911543) or dual JAK1/2 inhibitors (ruxolitinib or baricitinib) at 1 μM, followed by IFN-γ/TNF-α (10 ng/ml each) for 72 h. **D** Relative viability of HT-29 cells pretreated for 1 h with DMSO, baricitinib (1 μM) or a cocktail of apoptosis/necroptosis inhibitors (necrostatin-1s (10 μM), GSK′872 (10 μM), NSA (1 μM) and zVAD (20 μM)) and increasing concentrations of *N*-acetylcysteine (NAC, 0.1, 1 and 10 mM), followed by (left) IFN-γ/TNF-α (10 ng/ml each) or (right) H_2_O_2_ (1 mM) for 72 h. Data were mean ± SEM of *n* = 3 independent experiments. *p < 0.05 and ***p < 0.001 (two-way ANOVA with Tukey’s multiple comparisons test). NT non-treated, nec-1s necrostatin-1s, G′872 GSK′872, CASP9-i caspase-9 inhibitor, UPA upadacitinib, BARI baricitinib, RUXO ruxolitinib, BMS BMS-911543, NAC *N*-acetylcysteine.

Our initial characterisation revealed that IFN-γ/TNF-α-induced killing involved cleavage of caspase-9 (Fig. 1D), indicative of mitochondrial apoptosis. This pathway can be engaged independently of death receptor signalling, for instance by IFN-γ-mediated upregulation of PUMA [45]. However, a caspase-9 inhibitor Z-LEHD, while reducing caspase-3 activity, did not improve the viability of dually treated cells (Fig. 5A). In line with this, concomitant depletion of BAX/BAK1—critical but mutually redundant regulators of mitochondrial apoptosis [46]—had no rescuing effect (Fig. 5B) unlike JAKinibs, which restored cell viability also in the context of BAX/BAK1 co-repression (Fig. 5C). Although reactive oxygen species (ROS) were previously implicated in IFN-γ/TNF-α-induced cytotoxicity [47], *N*-acetylcysteine had no protective effect, even when apoptosis and necroptosis were co-inhibited (Fig. 5D). Collectively, TNF-α synergised with IFN-γ to kill IECs through a non-canonical TNFR1-mediated pathway that was independent of RIPK1/3, MLKL or caspase activity.

### Caspase-8 nonenzymatic function supports lethal JAK1/2-STAT1 signalling

**Fig. 6:**
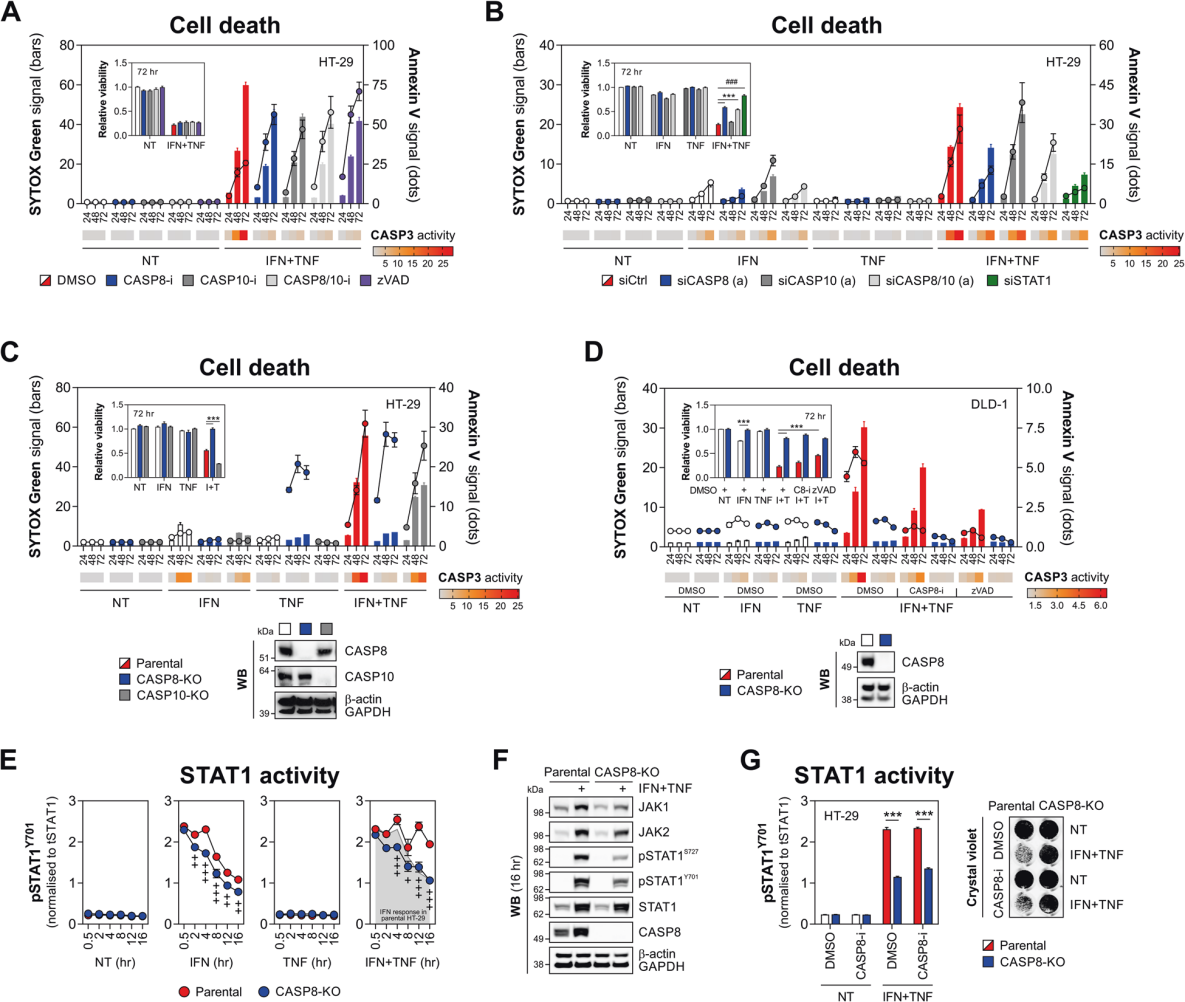
Caspase-8 nonenzymatic function is required for IEC killing by IFN-γ/TNF-α. **A** HT-29 cells were pretreated for 1 h with vehicle (DMSO), caspase-8 inhibitor (Z-IETD, 10 μM), caspase-10 inhibitor (Z-AEVD, 10 μM) or zVAD (20 μM), followed by IFN-γ/TNF-α (10 ng/ml each). SYTOX Green/Annexin V signals and caspase-3 activity (heatmap) were recorded at indicated times, and relative viability was measured at 72 h (inner graph). **B** HT-29 cells were transfected with a nontargeting siRNA (siCtrl), siCASP8, siCASP10 or a positive control siRNA (siSTAT1), followed by IFN-γ/TNF-α (10 ng/ml each) for 72 h. SYTOX Green/Annexin V signals and caspase-3 activity (heatmap) were recorded at indicated times, and relative viability was measured at 72 h (inner graph). For both targets, siRNA’s 'a' were used. **C** Parental, CASP8 or CASP10 knockout HT-29 cells were treated with IFN-γ/TNF-α (10 ng/ml each). SYTOX Green/Annexin V signals and caspase-3 activity (heatmap) were recorded at indicated times, and relative viability was measured at 72 h (inner graph). Western blot validation of CASP8/10 knockout (bottom). **D** Parental or CASP8 knockout DLD-1 cells were pretreated for 1 h with vehicle (DMSO), caspase-8 inhibitor (Z-IETD, 10 μM) or zVAD (20 μM), followed by IFN-γ/TNF-α (10 ng/ml each). SYTOX Green/Annexin V signals and caspase-3 activity (heatmap) were recorded at indicated times, and relative viability was measured at 72 h (inner graph). Western blot validation of CASP8 knockout (bottom). **E** STAT1 activity was measured by HTRF assay in parental and CASP8 knockout HT-29 treated with IFN-γ and/or TNF-α (10 ng/ml each) for indicated times. **F** Western blot analysis of JAK1/2, STAT1 (total, Y701- and S727-phosphorylated) and CASP8 expression in parental and CASP8 knockout HT-29 treated with IFN-γ/TNF-α (10 ng/ml each) for 16 h. **G** STAT1 activity at 16 h measured by HTRF assay (left), and crystal violet staining at 72 h (right) in parental and CASP8 knockout HT-29 pretreated for 1 h with DMSO or caspase-8 inhibitor (Z-IETD, 10 μM), followed by IFN-γ/TNF-α (10 ng/ml each). Data were mean ± SEM of *n* = 3 independent experiments. ***p < 0.001 (two-way ANOVA with Tukey’s multiple comparisons test), ^+^*p* < 0.05, ^++^*p* < 0.01 and ^+++^*p* < 0.001 (two-way ANOVA with Bonferroni’s multiple comparisons test vs. time point-matched parental HT-29 cells), ^###^*p* < 0.001 (unpaired *t*-test). NT non-treated, C8-i/CASP8-i caspase-8 inhibitor, CASP10-i caspase-10 inhibitor, KO knockout, I + T IFN + TNF.

Given cleavage of caspase-8/10 in our model (Fig. 1D) and their reported nonredundant cell death functions [48, 49], we applied inhibitors of caspase-8 (Z-IETD) and caspase-10 (Z-AEVD), and found that even caspase-8/10 co-inhibition failed to rescue HT-29 cells from IFN-γ/TNF-α (Fig. 6A), which was functionally consistent with the effect of zVAD (Fig. 4B–D). Apart from its canonical protease role in apoptosis, caspase-8 can also regulate various inflammatory responses through its nonenzymatic scaffold function [50–52]. Unlike caspase inhibition, we found that siRNA-mediated depletion of caspase-8, but not caspase-10, conferred resistance of HT-29 cells to IFN-γ/TNF-α (Figs. 6B and S4), an effect fully recapitulated in CASP8-KO HT-29 and DLD-1 cells (Fig. 6C, D). Together, this suggested caspase-8 played a key nonenzymatic role in modulation of IEC death driven by the JAK1/2-STAT1 pathway.

To explore this potential regulatory cross-talk, we first monitored STAT1^Y701^ phosphorylation in parental and CASP8-KO HT-29 cells. While IFN-γ and IFN-γ/TNF-α induced equivalent STAT1 activation in the parental line at early stages, IFN-γ/TNF-α drove sustained STAT1^Y701^ phosphorylation at 12–16 h compared to IFN-γ (Fig. 6E). This synergistic response was impaired in CASP8-KO cells, in which STAT1 activity returned to the levels detected during a nonlethal stimulation of the parental line with IFN-γ (Fig. 6E). In addition, caspase-8 knockout reduced IFN-γ/TNF-α-driven induction of other key components of the JAK1/2-STAT1 pathway, including JAK1, JAK2 and STAT1^S727^ phosphorylation (Fig. 6F). Of note, caspase-8 itself was also upregulated by IFN-γ/TNF-α (Fig. 6F), suggesting a positive feedback loop. Consistent with its lack of impact on cell survival (Fig. 6A), the caspase-8 inhibitor Z-IETD had no inhibitory effect on STAT1 activation (Fig. 6G). Collectively, the nonenzymatic function of caspase-8 was essential for synergistic IEC killing by IFN-γ/TNF-α, at least in part by supporting lethal activation of JAK1/2-STAT1 signalling.

### Activation of the CASP8-JAK1/2-STAT1 cell death module in human colonic biopsies and organoids

**Fig. 7:**
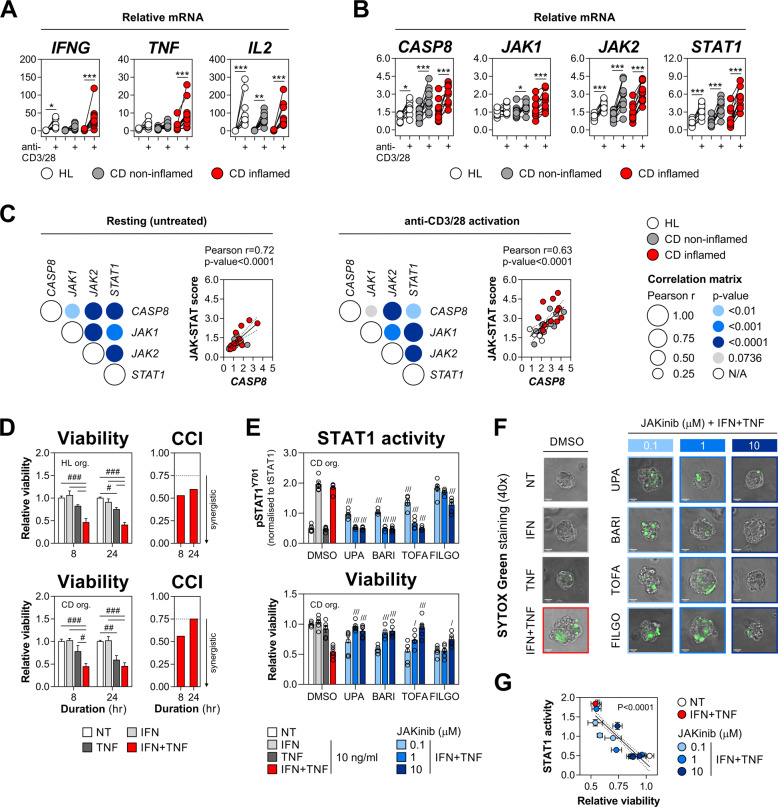
Activation of the CASP8-JAK1/2-STAT1 cell death module in human colonic biopsies and organoids. **A**–**C** Colonic biopsies isolated from healthy (HL) individuals (*n* = 10) or patients with inactive/non-inflamed (*n* = 11) and active/inflamed (*n* = 11) Crohn’s disease (CD) were incubated ex vivo with anti-CD3/28 (5 μg/ml each) for 18 h. Relative mRNA expression of **A** *IFNG, TNF* and *IL2*, and **B** *JAK1*, *JAK2*, *STAT1* and *CASP8* measured by RT-qPCR. **C** Correlation between relative mRNA levels of the indicated genes or the JAK-STAT score in resting (left) and anti-CD3/28-stimulated biopsies (right). **D** Relative viability of primary human colonic organoids derived from HL (*n* = 3 lines) or CD (*n* = 3 lines) colonic biopsies, and stimulated with IFN-γ and/or TNF-α (10 ng/ml each) for the indicated times. Coefficient of cytokine interaction (CCI) calculated per time-point (right). **E**–**G** Primary human colonic organoids derived from CD colonic biopsies (*n* = 3 lines) were pretreated for 1 h with vehicle (DMSO), JAK1-selective compound (upadacitinib and filgotinib), JAK1/2 inhibitor (baricitinib) or pan-JAK inhibitor (tofacitinib) at 0.1, 1 or 10 μM, followed by IFN-γ and/or TNF-α (10 ng/ml each) for the indicated times. **E** STAT1 activity was measured by HTRF assay at 2 h (top) and relative viability at 8 h (bottom) of cytokine treatment. **F** SYTOX Green staining of a select organoid line at 8 h of cytokine treatment. **G** Pearson correlation between relative viability and STAT1 activity. Data were mean ± SEM. *p < 0.05, **p < 0.01 and ***p < 0.001 (two-way, repeated-measures ANOVA with Bonferroni’s multiple comparisons test), ^#^*p* < 0.05, ^##^*p* < 0.01 and ^###^*p* < 0.001 (two-way ANOVA with Tukey’s multiple comparisons test), ^/^*p* < 0.05, ^///^*p* < 0.001 (one-way ANOVA with Dunnett’s multiple comparisons test vs. DMSO plus IFN-γ/TNF-α). NT non-treated, JAKinib JAK inhibitor, UPA upadacitinib, BARI baricitinib, TOFA tofacitinib, FILGO filgotinib, org. organoids, CCI coefficient of cytokine interaction.

To evaluate the clinical relevance of the above findings, we first studied the expression of the key drivers of IFN-γ/TNF-α-induced IEC death (CASP8, JAK1/2 and STAT1) in human mucosal biopsies following anti-CD3/28 stimulation. This ex vivo T cell activation protocol generates a Th1-type immune response reminiscent of the CD-associated cytokine profile and was shown to promote the killing of intestinal enteroids [2, 24]. In line with this, anti-CD3/28-stimulated colonic biopsies expressed high levels of *IFNG*, *TNF* and *IL2* compared to the resting tissue, and this induction was most prominent in inflamed CD samples (Fig. 7A). Similar to the cell line-based results, we detected upregulation of *CASP8, JAK1/2* and *STAT1* following T cell activation irrespective of the disease state (Fig. 7B). While we could not infer causation between the induction of the Th1-type cytokines and the CASP8-JAK1/2-STAT1 module, the expression of the four cell death driver genes was positively intercorrelated in resting and T cell-activated biopsies (Fig. 7C). Under both these conditions, *CASP8* expression itself was positively correlated with the net JAK-STAT score (Fig. 7C).

To test the causative role of JAK1/2-STAT1 signalling in a primary 3D model of IEC death, we utilised colonic organoids expanded from epithelial crypts of healthy individuals (*n* = 3) or patients with CD (*n* = 3). Similar to 2D cell line cultures, colonic organoids retained synergistic sensitivity to IFN-γ/TNF-α, although we did note that killing occurred at an accelerated rate, with CD-derived organoids being equally susceptible to TNF-α alone by 24 h (Fig. 7D). Using previously validated JAKinibs, we found that upadacitinib, baricitinib and a pan-JAK inhibitor tofacitinib dose-dependently rescued CD-derived colonic organoids from IFN-γ/TNF-α, while having a parallel inhibitory effect on STAT1 activation (Fig. 7E). By contrast, filgotinib—which was marginally protective in HT-29 cells—showed similar subpar activity in the 3D model of intestinal damage. Consistent with their effects on STAT1 activation and survival, JAKinibs prevented a cytokine-triggered collapse of organoid structure and SYTOX Green cell death staining (Fig. 7F), and their protective effect was negatively correlated with the remaining STAT1 activity (Fig. 7G). In sum, we report a correlated induction of the lethal CASP8-JAK1/2-STAT1 module during Th1-type immune response in human colonic mucosal biopsies and validate the efficacy of JAKinibs in a primary organoid model of IFN-γ/TNF-α-driven intestinal damage.

## Discussion

The Th1-type cytokines IFN-γ and TNF-α are associated with increased IEC death and disruption of gut epithelial barrier function in IBD [1, 2]. The phenomenon of synergistic killing by IFN-γ/TNF-α was first reported in cancer cells in 1983 [53] and extends to multiple non-transformed cell types [37, 54, 55], including IECs [18–21, 56] and most recently macrophages in the context of COVID-19 [57]. Yet, the mechanisms underpinning this synergistic lethal response in IECs remain incompletely understood. Considering the emerging causal role of IFN-γ in IEC death [20, 23–26] and widespread resistance to anti-TNFs in CD [16, 17], we reasoned this under-looked synergy might offer insights into tractable effectors of immune-mediated tissue damage in CD. Indeed, inhibition of synergistically IFN-γ/TNF-α-induced MLCK1 trafficking in IECs was recently leveraged to restore barrier function in experimental IBD [58]. Through unbiased kinome RNAi and drug repurposing screens we identified JAK1/2 as the sole kinase drivers of IEC death triggered by IFN-γ/TNF-α. This unique form of inflammatory cell death, while executed by the CASP8-JAK1/2-STAT1 module downstream of TNFR1, proved insensitive to combined inhibition of apoptosis, necroptosis and ROS, and is therefore likely mediated via multiple redundant downstream effectors. Having validated the potential disease relevance of these discoveries in CD-derived colonic mucosal biopsies and primary organoids, we propose that inhibition of this unique mode of IEC death may underpin part of the clinical efficacy of both anti-TNFs and JAKinibs in IBD.

Our data suggest lethal crosstalk between proximal TNFR1 signalling and the JAK1/2-STAT1 pathway. TNF-α was previously shown to induce the assembly of a TNFR1-STAT1 complex, which impaired NF-κB activity and sequestered phosphorylated STAT1^Y701^ in the cytoplasm [59]. In macrophages, this inhibitory TNFR1-STAT1 interaction was relieved by IFN-γ [60]. Although NF-κB is considered pro-survival, chronic NF-κB signalling can sensitise intestinal crypts to TNF-α-driven apoptosis [61]. While the above crosstalk and this counterintuitive aspect of NF-κB signalling merit further investigation, we found that synergistic IEC death required STAT1 activation. Indeed, increased STAT1 activity, rather than its upregulation, was critical for the IFN-γ/TNF-α-induced killing of β cells [37]. Other studies suggest that, while STAT1^Y701^ phosphorylation facilitates STAT1–TRADD interaction, TNF-α-mediated toxicity requires primarily the S727 residue of STAT1 [59, 62, 63]. It is, therefore, possible that IFN-γ/TNF-α are synergistically lethal as a result of chronic STAT1 and NF-κB signalling mediated by IFN-γ-driven inhibition of the TNFR1-STAT1 complex and TNF-α-enhanced binding of TRADD to RIPK1 and/or TRAF2 [59, 60]. The latter could decrease the propensity of TRADD to engage (and inhibit) STAT1 [64], facilitating STAT1 activation and thus IEC death. Coincidentally, elevated nuclear NF-κB/p65 staining [61] and STAT1 phosphorylation [33] were detected in inflamed CD mucosa.

Multiple small-molecule JAKinibs are currently under clinical development for the treatment of IBD. However, little is known about their direct effects on IEC functions [65]. Recently, pan-JAK or JAK1/2 inhibitors were shown to rescue mouse intestinal organoids from IFN-γ-induced barrier dysfunction [66] or killing [25]. Here, we extend these observations by showing that JAKinibs, including JAK1-selective compounds, can protect human IECs from TNF-α-mediated cytotoxicity. Except for filgotinib, all tested JAKinibs dose-dependently rescued colonic cell lines and primary organoids from IFN-γ/TNF-α. This protective effect was directly linked to inhibition of STAT1^Y701^ phosphorylation, with upadacitinib being the most potent JAKinib. Of note, upadacitinib proved efficacious in patients with 'difficult-to-treat' CD (96% failed or intolerant to one or more TNF-α biologics) [67], while the anti-IFN-γ antibody fontolizumab displayed limited efficacy in active CD [2]. These contrasting clinical outcomes likely stem from the capacity of JAKinibs to affect multiple cytokine-dependent pathways [65]. Conceptually, during flares IFN-γ priming may induce maladaptive trained immunity that could sensitise IECs to future insults [22, 68–70]. This would likely be inhibited by JAKinibs but not through IFN-γ blockade. In fact, IBD-derived intestinal organoids retain transcriptional memory of inflammation [71], which may explain the increased susceptibility of CD-derived organoids to TNF-α in our study.

Similar to synergistic mucosal barrier disruption [19], we found that killing of IECs by IFN-γ/TNF-α was independent of caspase activation. Depletion of apoptotic effectors within IECs can, however, promote TNF-α-mediated necroptosis via RIPK3 [9, 10], while IFN-γ/TNF-α-driven killing itself can also be executed by RIPK1/MLKL [55]. Unexpectedly, IFN-γ/TNF-α-induced IEC death was not compromised by co-inhibition of apoptosis and necroptosis in our study. This is in contrast to findings in β cells, whereby IFN-γ/TNF-α-driven killing proceeds primarily via mitochondrial apoptosis [47, 72, 73], suggesting cell type-specific differences in the execution of this synergistic lethal response. It also implies that, unlike non-physiological sensitisers such as translational inhibitors or SMAC mimetics [41], IFN-γ sensitises IECs to TNF-α independently of canonical cell death signalling. A recent study found that loss of autophagy genes including *Atg16l1* conferred hypersensitivity of myeloid cells to IFN-γ via TNF-α signalling [74]. Coincidentally, *ATG16L1* gene harbours a CD risk allele [34], and *Atg16l1* deficiency was shown to amplify TNF-α-driven IEC death [75]. Considering this and delayed killing of IECs by IFN-γ/TNF-α, this synergistic lethal response may be in part supported by progressive downregulation of cytoprotective effectors like *ATG16L1*.

Intriguingly, we found that synergistic killing of IECs by IFN-γ/TNF-α required a nonenzymatic caspase-8 scaffold. Catalytically dead caspase-8 can act as a nucleation platform for ASC, which facilitates pyroptosis of IECs when apoptotic and necroptotic signalling are compromised [76, 77]. We previously observed that IFN-γ/TNF-α-treated IECs released HMGB1 (unpublished data), a DAMP known to induce ASC-dependent pyroptosis via lysosomal damage [78]. Coincidentally, lysosome-associated cell death driven by catalytically inactive caspase-8 was recently reported in cancer cells [79]. While this may constitute a backup mechanism of IFN-γ/TNF-α-induced killing, we found that the scaffold function of caspase-8 facilitated activation of lethal JAK1/2-STAT1 signalling. Although STAT1-dependent control of caspases is known [62], caspase-mediated modulation of STAT1 function can equally occur. In particular, both genetic and catalytic inhibition of caspase-8 blocked STAT1^Y701^ phosphorylation during CD95 stimulation [80]. In our study, however, Z-IETD failed to impact STAT1 activation, suggesting context-dependent regulation of STAT1 by caspase-8. A caspase-8 scaffold was also found critical for inflammatory signalling downstream of the TRAIL receptor [50, 51] or TLR3 [52]. Both these processes relied, however, on FADD and RIPK1, which are not required for IFN-γ/TNF-α-induced death of IECs (unpublished data). Most recently, IFN-γ/TNF-α synergy was causally implicated in macrophage PANoptosis in the context of COVID-19 [57]. Specifically, the authors identified NO as an actionable effector of the FADD-STAT1-IRF-1 pathway utilised by IFN-γ/TNF-α for synergistic macrophage killing. However, we (unpublished data) and others [81] found that iNOS inhibition fails to protect IECs from IFN-γ/TNF-α, suggesting other mechanisms are at play in these cells.

In sum, we show that synergistic killing of IECs by IFN-γ/TNF-α, while executed by the CASP8-JAK1/2-STAT1 module, is independent of canonical cell death signalling. This inherent plasticity could represent an under-looked therapeutic barrier in achieving mucosal healing in CD. On the other hand, exquisite sensitivity of the cell death phenotype to JAKinibs can explain part of their clinical efficacy, by underscoring a direct protective effect on IECs. While deleterious if unchecked, functional IFN-γ/TNF-α synergy can also generate robust antiviral immunity [82]. Of note, multiple viruses evade immunogenic death of host cells by encoding suppressors of caspase-8 or RIPK1/3 [83]. The synergistic lethal interaction between IFN-γ/TNF-α might have therefore evolved as an ultimate defence mechanism allowing for the elimination of infected cells once a critical inflammatory set point is reached. Intriguingly, patients with early-onset IBD caused by a deficiency in caspase-8, a scaffold required for synergistic activation of JAK1/2-STAT1 and IEC death as identified herein, were also increasingly susceptible to viral infections [84].

## Materials and methods

### Cell culture

Unless specified otherwise, cell lines were purchased from the American Type Culture Collection, and cultured in the indicated media supplemented with 10% FBS, 100 IU/ml penicillin and 0.1 mg/ml streptomycin: HT-29 cells (HTB-38, McCoy’s 5 A), COLO205 (CCL-222, RPMI 1640), DLD-1 cells (CCL-221, RPMI 1640), HCA-7 (06061902-1VL Sigma-Aldrich, DMEM), HCT-116 (CCL-247, DMEM), HCT-15 (CCL-225, RPMI 1640), LoVo (CCL-229, Ham’s F-12K), RKO (CRL-2577, EMEM), SW-48 (CCL-231, L-15), SW-948 (CCL-237, L-15), while L-WRN cells (CRL-3276, DMEM) required additional 0.5 mg/mL G-418 and 0.5 mg/mL hygromycin B. Parental, CASP8 and CASP10 knockout HT-29 cells [85] were a kind gift from Prof. John Silke (Walter and Eliza Hall Institute of Medical Research, Parkville, Victoria, Australia), and were cultured in complete RPMI 1640 medium. All cell lines were routinely screened for mycoplasma contamination using MycoAlert Mycoplasma Detection Kit (LT07-118, Lonza).

### Recombinant cytokines, antibodies and reagents

Recombinant human cytokines IFN-γ (300-02) and TNF-α (300-01 A) were from Peprotech. CellTiter-Glo (G7573) and Caspase-Glo 3/7 (G8091) were from Promega. For western blotting, the following antibodies were used: β-actin (1:5000, sc-47778, SCBT), GAPDH (1:80,000, AM4300, Ambion), caspase-3 (1:1000, 9662, CST), cleaved caspase-3 (1:1000, 9661, CST), caspase-8 (1:1000, 9746, CST), caspase-9 (1:1000, 9508, CST), cleaved caspase-9 (1:1000, 7237, CST), caspase-10 (1:1000, M059-3, MBL), MLKL (1:500, 184718, Abcam), p-MLKL S358 (1:500, 187091, Abcam), BAX (1:1000, 2772, CST), BAK1 (1:1000, 3814, CST), STAT1 (1:2000, 9172, CST), p-STAT1 Y701 (1:2000, 9167, CST), p-STAT1 S727 (1:1000, 9177, CST), JAK1 (1:1000, 3332, CST), JAK2 (1:1000, 3230, CST), TNFR1 (1:1000, 3736, CST) goat anti-rabbit Ig-HRP (P0448, Dako) and rabbit anti-mouse Ig-HRP (P0260, Dako). For T cell activation studies, purified NA/LE mouse anti-CD3 (555336) and anti-CD28 (555725) were from BD Biosciences. For cell death studies, necrostatin-1s (20924, Cayman Chemical), GSK'872 (530389, Calbiochem), NSA (20844, Cayman Chemical) as well as the following caspase inhibitors were used: Z-VAD-FMK (14463, Cayman Chemical), Z-IETD-FMK (FMK007, R&D Systems), Z-LEHD-FMK (FMK008, R&D Systems) or Z-AEVD-FMK (14987, Cayman Chemical). To sensitise cells to TNF-α, we used the following compounds: cycloheximide (C7698, Sigma-Aldrich), BV6 (S7597, Selleckchem) and IKK-16 (S2882, Selleckchem). The following JAK inhibitors were used: baricitinib (S2851, Selleckchem), upadacitinib (HY-19569, Insight Biotechnology), filgotinib (S7605, Selleckchem), BMS-911543 (21088, Cayman Chemical), fedratinib (S2736, Selleckchem), ruxolitinib (S1378, Selleckchem) and tofacitinib (CP-690550, Cayman Chemical). Unless specified otherwise, all other reagents were from Sigma-Aldrich.

### End-point cell viability and cell death assays

All assays were performed in a 96-well plate format and analysed using Synergy 2 microplate reader (BioTek) as previously described [21]. For crystal violet staining, following 10-min fixation with ice-cold methanol at −20 °C cells were stained with crystal violet solution (0.5% crystal violet in 25% methanol) for 10 min at RT. Washed and air-dried plates were scanned using the Odyssey 9120 imaging system (LI-COR), and digital images were processed in ImageJ software. For some experiments, crystal violet in fixed cells was re-solubilised in DMSO and its absorbance was measured at 590 nm. For measurement of cell viability and caspase-3/7 activity, CellTiter-Glo and Caspase-Glo 3/7 assays were used according to the manufacturer’s instructions. In addition to plate-based assays, cell viability and caspase-3 activation was also monitored by flow cytometry as previously described [21]. Briefly, following cytokine treatment HT-29 cells were trypsin-harvested, stained with Fixable Viability Stain 660 (564405, BD Biosciences, 1:2000 in PBS) for 15 min at RT and washed with FACS buffer (2% FBS in PBS). After 20-min incubation with Fixation-Permeabilization reagent (554722, BD Biosciences) at 4 °C, cells were washed in BD Perm/Wash (554723, BD Biosciences) and stained for 30 min with active caspase-3 antibody (561011, BD Biosciences, 1:5 in BD Perm/Wash) at 4 °C. Following washing in BD Perm/Wash, cells were resuspended in FACS buffer with 2 mM EDTA, run on FACSCelesta (BD Biosciences) and analysed with FlowJo v10 software.

### Real-time cell death assays

All assays were performed in a 96-well plate format and analysed using a Synergy 2 microplate reader (BioTek). For detection of Annexin V signal, RealTime-Glo Annexin V Apoptosis assay (JA1001, Promega) was used according to the manufacturer’s instructions. For detection of SYTOX Green cell death signal, cells were continuously incubated with a cell-impermeable SYTOX Green dye (2.5 μM, S7020, Thermo Fischer), whose fluorescent intensity was measured at 485/530 nm as previously described [86]. For detection of caspase-3 activity, cells were continuously incubated with Ac-DEVD-AMC (10 μM, HY-P1003, Medchem Express), whose fluorescent intensity was measured at 330/460 nm as previously described [86]. Both SYTOX Green cell death signal and caspase-3 activity were normalised to the total DNA fluorescence [87] measured at 330/460 nm by incubating cells for 30 min with Hoechst 33342 (10 μg/ml in PBS, H3570, Thermo Fischer). For all three cell death assays, data were expressed as a fold change over the non-treated control group.

### Evaluation of synergy between IFN-γ and TNF-α

To evaluate the synergistically inhibitory effect of IFN-γ and TNF-α on cell survival, a coefficient of cytokine interaction (CCI) was introduced based on the analogy to studies on drug interactions [88]. CCI was calculated as follows: I + T÷(I×T), where I + T, I and T correspond to viability after dual IFN-γ/TNF-α treatment, or individual stimulations with IFN-γ or TNF-α, respectively. CCI values indicate synergistic (<1), additive (=1) or antagonistic (>1) relationships. For the purpose of this study, CCI <0.75 was considered as a synergistic cytokine interaction.

### Kinome RNAi screen

The kinome RNAi screen was performed in duplicate in 384-well format using the kinome ON-TARGETplus (OTP) SMARTpool human siRNA library (pools of four siRNAs per target) extracted from the genome-wide OTP SMARTpool human siRNA library (Dharmacon), and targeting 659 human genes annotated as 'kinases' at the Functional Genomics Core facility, Sanford Burnham Prebys Medical Discovery Institute, La Jolla, CA, USA. The library was spotted in black-clear bottom 384-well plates (781092, Greiner) together with positive and negative controls at 0.5 pmol/well and reverse transfected into HT-29 cells by adding of 10 μl of Opti-MEM:RNAiMAX mixture (10:1, Life Technologies) and cells to a density of 1000 cells/well. Cells were incubated for 48 h to allow protein decay, treated with IFN-γ and TNF-α (10 ng/ml each) for 72 h, after which cell viability was read using CellTiter-Glo. Raw viability data were normalised to non-silencing control wells in the absence of cytokines included in every plate and used to extract a normalised average and robust *Z*-score for each target.

### Drug library screen

The drug library screen was performed in triplicate in 384-well format using a manually curated library of FDA-approved drugs at the Functional Genomics Core facility, Sanford Burnham Prebys Medical Discovery Institute, La Jolla, CA, USA. HT-29 cells were seeded to a density of 5000 cells/well for 24 h. Cells were pretreated with 10 μM of each drug for 1 h, followed by stimulation with IFN-γ and TNF-α (10 ng/ml each) for 72 h, after which cell viability was read using CellTiter-Glo. Raw viability data were normalised to vehicle (DMSO) control wells in the absence of cytokines included in every plate and used to extract a normalised average and robust *Z*-score for each drug.

### Human studies

The collection and use of colonic pinch biopsies was ethically approved by the Clinical Research Ethics Committee of the Cork Teaching Hospitals (CREC). All patients provided informed consent in agreement with the Declaration of Helsinki. The biopsies were collected from adult non-IBD patients or from patients with CD at Cork University Hospital or Mercy University Hospital. All non-IBD patients were comprised of otherwise healthy individuals undergoing investigation of symptoms such as weight loss, anaemia or altered bowel habit. All patients with CD had a confirmed diagnosis as evidenced by previous histopathological findings. The active and inactive disease was differentiated by the attending physician based on endoscopic and histological criteria.

### Primary human colonic organoid culture

A detailed protocol of crypt isolation from colonic pinch biopsies and subsequent organoid culture was previously published [21] and followed without alterations. Live transmission light microscopy imaging was done using the EVOS FL inverted microscope (Invitrogen). Acquired digital images were processed in ImageJ-win64, which included cropping around the area of interest, brightness/contrast adjustment using Enhance Local Contrast (CLAHE) plugin and insertion of 50 μm scale bars.

### SYTOX Green organoid imaging

Organoids were seeded as described above at 5000 cells per well and treated as indicated in the figure legends. Thirty minutes before completion of the treatment SYTOX Green (2.5 μM in proliferation medium) was added directly to the wells, and organoids were returned to the incubator till the end-point. After the treatment, the medium was removed and replaced with a fresh proliferation medium, and samples were imaged immediately. All imaging was performed on live organoids using the EVOS FL inverted microscope and a 40x Plan Fluor objective (Invitrogen). For each image phase, contrast transmission and GFP channels were acquired, and the overlayed image was exported as an RGB file. GFP LED light cube illumination intensity was kept consistent between images for each experiment. Image processing was performed using ImageJ-win64. Images were cropped around the region of interest, processed using the Enhance Local Contrast (CLAHE) plugin and a scale bar was inserted.

### T cell activation in colonic biopsies

For T cell activation studies, colonic pinch biopsies were collected in Biopsy Collection medium: advanced DMEM/F12 (12634010, Gibco) supplemented with 10% FBS, 100 IU/ml penicillin, 0.1 mg/ml streptomycin, 100 μg/mL gentamicin, 10 mM HEPES (15630080, Gibco), 2 mM Glutamax (35050061, Gibco) and 1 × Amphotericin B (11526481, HyClone). Upon arrival, the biopsies were transferred to 24-well plates containing 1 ml/well of fresh Biopsy Collection medium ± anti-human CD3/28 (5 μg/ml each), and incubated for 18 h in humidified 5% CO_2_ atmosphere at 37 °C. Afterwards, the biopsies were processed immediately for RNA isolation.

### siRNA-mediated gene knockdown

HT-29 cells were reverse transfected with siRNA (25 nM) for 48 h using Lipofectamine RNAiMAX (13778150, Invitrogen) as previously described [21]. The following Silencer Select siRNAs were purchased from Thermo Fisher: siCASP8-a (s2427), siCASP8-b (s2426), siCASP10-a (s534109), siCASP10-b (s534111) and a related nontargeting negative siCtrl (4390843). The following ON-TARGETplus SMARTpool siRNAs were purchased from Horizon Discovery: siJAK1 (L-003145-00-0005), siJAK2 (L-003146-00-0005), siSTAT1 (L-003543-00-0005), siTNFR1 (L-005197-00-0005), siTNFR2 (L-003934-00-0005), siBAX (L-003308-01-0005), siBAK1 (L-003305-00-0005), siRIPK1 (L-004445-00-0005), siRIPK3 (L-003534-00-0005), siZBP1 (L-014650-00-0005) and a related nontargeting negative siCtrl (D-001810-10-20). Knockdowns were validated by western blotting or RT-qPCR.

### CRISPR/Cas9-mediated gene knockout

Alt-R CRISPR-Cas9 crRNAs targeting JAK1 (TCAACGGTGATGGTGCGATT), JAK2 (TATCGGCATGGAATATCTCG), STAT1 (TGTGATAGGGTCATGTTCGT) as well as ATTO 550-labelled Alt-R CRISPR-Cas9 tracrRNA (1075928) were from IDT. Single-cell knockout clones of DLD-1 cells were generated by reverse transfection of gRNA-Cas9 RNP complexes using Lipofectamine CRISPRMAX (CMAX00001, Invitrogen) as previously described [21]. Single-cell knockout clones of HT-29 cells were generated by electroporation of gRNA-Cas9 RNP complexes using the Neon Transfection System (MPK10025, Invitrogen). Briefly, equimolar amounts of crRNA and tracrRNA were incubated in Duplex Buffer (11-01-03-01, IDT) for 5 min at 95 °C to generate 100 μM gRNA duplex. gRNA-Cas9 RNP complexes were prepared by incubating 1.2 μl of gRNA duplex, 1.7 μl of Alt-R Cas9 nuclease (1081058, IDT) and 2.1 μl of PBS for 20 min at RT. Per electroporation, 10 μl of freshly harvested HT-29 cell suspension (1.0 × 10^7^ cell/ml in PBS) were gently mixed with 2.5 μl of gRNA-Cas9 RNP complexes and electroporated at 1650 V, 10 ms, four pulses. Cells were immediately transferred into 0.5 ml of pre-warmed antibiotic-free, 10% FBS McCoy’s 5 A medium and incubated overnight in 24-well plates. The next day, cells were trypsin-harvested and single-cell sorted using FACSAria Fusion (BD Biosciences). Expanded clones were tested for knockout using Sanger sequencing with follow-up validation by western blotting. DLD1 CRISPR/Cas9 CASP8 knockout mixed population cell line was generated by retroviral infection with pLentiCRISPR with guide RNA AAGTGAGCAGATCAGAATTG, established following selection with 1 μg/ml puromycin, and clonally selected for the complete knockout by Western blot analysis as recently described [89].

### Western blotting

Sample preparation and western blotting were performed as previously described [21]. Cells, including detached ones, were freshly lysed in cold caspase lysis buffer (10 mM Tris–HCl pH 7.4, 10 mM NaCl, 3 mM MgCl_2_ and 1% NP-40) reconstituted with 0.25 mM AEBSF and Halt Protease and Phosphatase Inhibitor Cocktail (78440, Thermo Scientific). Cell debris was removed by high-speed centrifugation for 20 min at and 4 °C, and protein concentration was quantified with Pierce BCA Protein Assay Kit (23225, Thermo Scientific). Briefly, 25 μg of protein were resolved on Bolt 4–12% Bis-Tris Plus Gels (NW04125BOX, Invitrogen) under reducing conditions according to manufacturer’s instructions. After transfer onto PVDF membranes (IPVH00010, Millipore) using Criterion blotter (1704070, Bio-Rad), blocked membranes were incubated overnight at 4 °C with the indicated primary antibodies, washed and stained with secondary antibodies for 1 h at RT. Images were detected with WesternBright Quantum HRP substrate (K-12042-D10, Advansta) on LAS-3000 Imager (Fujifilm) and processed using ImageJ software with brightness/contrast adjustment applied to an entirely digital image if necessary.

### STAT1 activity assay

The activity of cellular STAT1 was measured using total STAT1 (63ADK096PEG) and phospho-STAT1^Y701^ (63ADK026PEG) HTRF (Homogeneous Time-Resolved Fluorescence) kits from Cisbio. Briefly, colonic organoids or 2 × 10^4^ HT-29 cells were seeded in 96-well plates and manipulated as indicated in the figures. After that, the medium was removed and samples were lysed fresh (organoids) or following storage at −80 °C (HT-29 cells) with 50 μl of provided 1× lysis buffer, processed according to manufacturer’s instructions and analysed using Synergy 2 microplate reader (BioTek) set to record fluorescence emission at 665 and 620 nm. STAT1 activity was calculated as a ratio of phospho-STAT1^Y701^ to total STAT1 signals and normalised to the average of all samples within an independent experiment.

### RNA isolation and gene expression analysis

RNA isolation, cDNA synthesis and gene expression analysis were performed as previously described [21]. Briefly, total RNA from cell lines was isolated using the RNeasy Mini kit (74106, Qiagen). For mucosal biopsies, following incubation with anti-CD3/28 antibodies, the biopsies were immediately placed in RLT buffer with 1% β-mercaptoethanol and disrupted using MagNA Lyser instrument and Green Bead tubes (03358941001, Roche) with two homogenisation cycles of 15 sec at 6500 rpm. Biopsy homogenates were processed using the RNeasy Mini kit and RNA Clean & Concentrator-5 kit (R1013, Zymo Research). Synthesis of cDNA was performed on 500 ng of RNA from cell lines (or 200 ng from biopsies) using Transcriptor Reverse Transcriptase (3531287001, Roche) and random hexamers. Gene expression analysis was done using Universal ProbeLibrary System (Roche) and Sensi Mix II Probe Kit reagent (BIO-83005, Bioline) according to the manufacturer’s instructions. Primers were purchased from Eurofins Genomics (Table S3). RT-qPCR reactions contained typically 2.5–10 ng of cDNA and were performed on LightCycler 480 instrument (Roche). Relative gene expression was analysed based on the 2^−ΔΔCT^ method [90] and normalisation to β-actin (cell lines) or the geometric mean of β-actin and GAPDH (mucosal biopsies). A JAK-STAT score was calculated by averaging the relative expression of *JAK1*, *JAK2* and *STAT1*, based on the analogy to an ISG score as previously described [75].

### Statistical analysis

Unless stated otherwise, data were presented as mean ± SEM and were tested for normality using SPSS Statistics v22 (IBM). Correlation analysis, between groups testing and IC50 calculations, were performed in GraphPad Prism v6 (GraphPad) as detailed in the figure legends. *P* < 0.05, *P* < 0.01 and *P* < 0.001 were considered statistically significant.

## Supplementary information

Supplementary Figure Legends

Supplementary Figure S1

Supplementary Figure S2

Supplementary Figure S3

Supplementary Figure S4

Supplementary Table S1

Supplementary Table S2

Supplementary Table S3

## Data Availability

Contact corresponding author for access to materials.
