# Supplementary material for: TNF-α synergises with IFN-γ to induce caspase-8-JAK1/2-STAT1-dependent death of intestinal epithelial cells
Source: Cell Death Dis. 2021 Sep 23;12(10):864. doi: 10.1038/s41419-021-04151-3 (PMC8459343; doi:10.1038/s41419-021-04151-3)
Supplement: Supplementary file 1 — Supplementary Figure Legends [file 41419_2021_4151_MOESM1_ESM.docx]

**Supplementary Figure Legends**

**TNF-α Synergises with IFN-γ to Induce Caspase-8-JAK1/2-STAT1-dependent Death of Intestinal Epithelial Cells**

Jerzy A. Woznicki ^1,*^, Nisha Saini ^1,*^, Peter Flood^1^, Subhasree Rajaram^1^, Ciaran M. Lee^1^, Panagiota Stamou^1^, Agnieszka Skowyra^1^, Milan Bustamante-Garrido^1^, Karine Regazzoni^1^, Nyree Crawford^4^, Simon S. McDade^4^, Daniel B. Longley^4^, Pedro Aza-Blanc^5^, Fergus Shanahan^1,2^, Syed A. Zulquernain^1,2^, Jane McCarthy^3^, Silvia Melgar^1^, Bradford L. McRae^6^, Ken Nally^1,7,†^

^1^ APC Microbiome Ireland, University College Cork, Cork, Ireland

^2^ Department of Medicine, University College Cork, Cork, Ireland

^3^ Department of Gastroenterology, Mercy University Hospital, Cork, Ireland

^4^ Centre for Cancer Research and Cell Biology, Queen's University Belfast, Belfast, UK

^5^ Sanford Burnham Prebys Medical Discovery Institute, La Jolla, CA 92037, USA

^6^ Immunology Discovery, Abbvie Bioresearch Center, Worcester, MA 01605, USA

^7^ School of Biochemistry and Cell Biology, University College Cork, Cork, Ireland

^*^ These authors contributed equally to this work

^†^ Corresponding author: [k.nally@ucc.ie](mailto:k.nally@ucc.ie)

**Fig S1. Dot plots of HT-29 cells stained for active caspase-3 and FVS660.** HT-29 cells were treated with IFN‑γ and/or TNF‑α (10 ng/ml each) for 72 hr, followed by staining for active caspase-3 and fixable viability dye FVS660, and analysis by flow cytometry. Representative dot plots and associated percentages of live/dying cells per each treatment group are indicated. The quantification of the indicated populations of live/dying cells is presented in Fig. 1C.

**Fig S2. JAK1/2 inhibition protects IECs from IFN‑γ/TNF‑α.** (**A**) HT‑29 cells were transfected with a non-targeting siRNA (siCtrl), siJAK1, siJAK2 or siSTAT1, followed by IFN‑γ/TNF‑α (10 ng/ml each) for 72 hr. SYTOX Green/Annexin V signals were recorded at indicated times, and relative viability was measured at 72 hr *(inner graph)*. Data are mean ± SEM of n=3 independent experiments. Western blot validation of target knockdown *(right)*. (**B**) Relative viability of HT-29 cells pre-treated for 1 hr with vehicle (DMSO), JAK1-selctive compound (filgotinib) or JAK2-selective compound (fedratinib) starting at 10 μM with 3-fold dilutions down to 1.52 nM, followed by IFN‑γ/TNF‑α (10 ng/ml each) for 72 hr. Data are mean ± SD of n=3 independent experiments. (**C**) Western blot analysis of STAT1 (total, Y701-phosphorylated) in HT-29 cells treated pre-treated for 1 hr with vehicle (DMSO) or indicated JAKinibs at 1 μM, followed by IFN‑γ/TNF‑α (10 ng/ml each) for 1 hr. (**D**) A JAK1 western blot image used in Fig. 3B prior to splicing. ****p* < 0.001 (two-way ANOVA with Tukey’s multiple comparisons test). NT – non-treated, JAKinib – JAK inhibitor, FILGO – filgotinib, UPA – upadacitinib, FEDE – fedratinib, BMS – BMS-911543, BARI – baricitinib, TOFA – tofacitinib.

**Fig S3. Perturbation of canonical cell death signalling in HT-29 cells.** (**A**) HT‑29 cells were transfected with a non-targeting siRNA (siCtrl), siRIPK1, siRIPK3, siZBP1 or a positive control siRNA (siSTAT1), followed, followed by IFN‑γ/TNF‑α (10 ng/ml each) for 72 hr. SYTOX Green/Annexin V signals and caspase-3 activity *(heatmap)* were recorded at indicated times, and relative viability was measured at 72 hr *(inner graph)*. RT-qPCR validation of target knockdown *(right)*. (**B**) Relative viability of HT-29 cells pre-treated for 1 hr with vehicle (DMSO), RIPK1 inhibitor (necrostatin-1s, 10 μM), RIPK3 inhibitor (GSK’872, 10 μM), MLKL inhibitor (NSA, 1 μM), dual JAK1/2 inhibitor (baricitinib, 10 μM) alone or in combination with a pan-caspase inhibitor (zVAD, 20 μM) along with cycloheximide (10 μg/ml), SMAC mimetic (BV6, 1 μM) or IKK inhibitor (IKK16, 1 μM), followed by TNF‑α (10 ng/ml) for 48 hr. Data are mean ± SEM of n=3 independent experiments. **p* < 0.05, ***p* < 0.01 and ****p* < 0.001 (two-way ANOVA with Tukey’s multiple comparisons test), ^###^p<0.001 (unpaired *t* test). NT – non-treated, nec-1s – necrostatin-1s, G’872 – GSK’872, CHX – cycloheximide, BARI – baricitinib.

**Fig S4. Loss of caspase-8 protects HT-29 cells from IFN‑γ/TNF‑α.** Relative viability of HT-29 cells transfected with a non-targeting siRNA (siCtrl), siCASP8 or siCASP10, followed by IFN‑γ/TNF‑α (10 ng/ml each) for 72 hr *(left)*. Relative mRNA expression *(middle)* and Western blot analysis *(right)* of CASP8 and CASP10 levels in siRNA-transfected HT‑29 cells. Two siRNA’s per target (labelled as “a” and “b”) were used. Data are mean ± SEM of n=3 independent experiments. **p* < 0.05, ***p* < 0.01 and ****p* < 0.001 (two-way ANOVA with Tukey’s multiple comparisons test). ^/^*p* < 0.05, ^///^*p* < 0.001 (one-way ANOVA with Dunnett’s multiple comparisons test vs. siCtrl). NT – non-treated.
