# Supplementary figures and images for: TNF-α synergises with IFN-γ to induce caspase-8-JAK1/2-STAT1-dependent death of intestinal epithelial cells
Source: Cell Death Dis. 2021 Sep 23;12(10):864. doi: 10.1038/s41419-021-04151-3 (PMC8459343; doi:10.1038/s41419-021-04151-3)

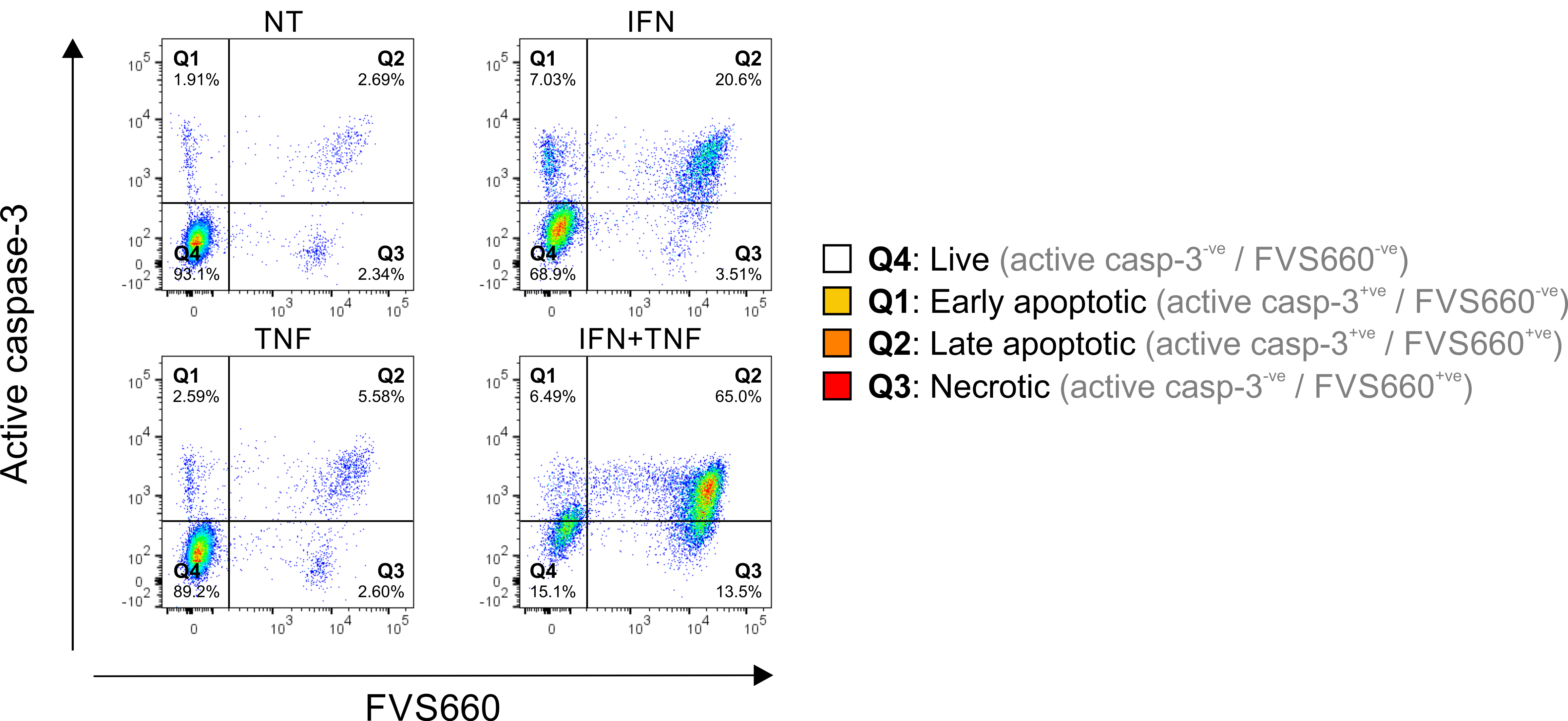

Supplement: Supplementary file 2 — Supplementary Figure S1 [file 41419_2021_4151_MOESM2_ESM.tif]

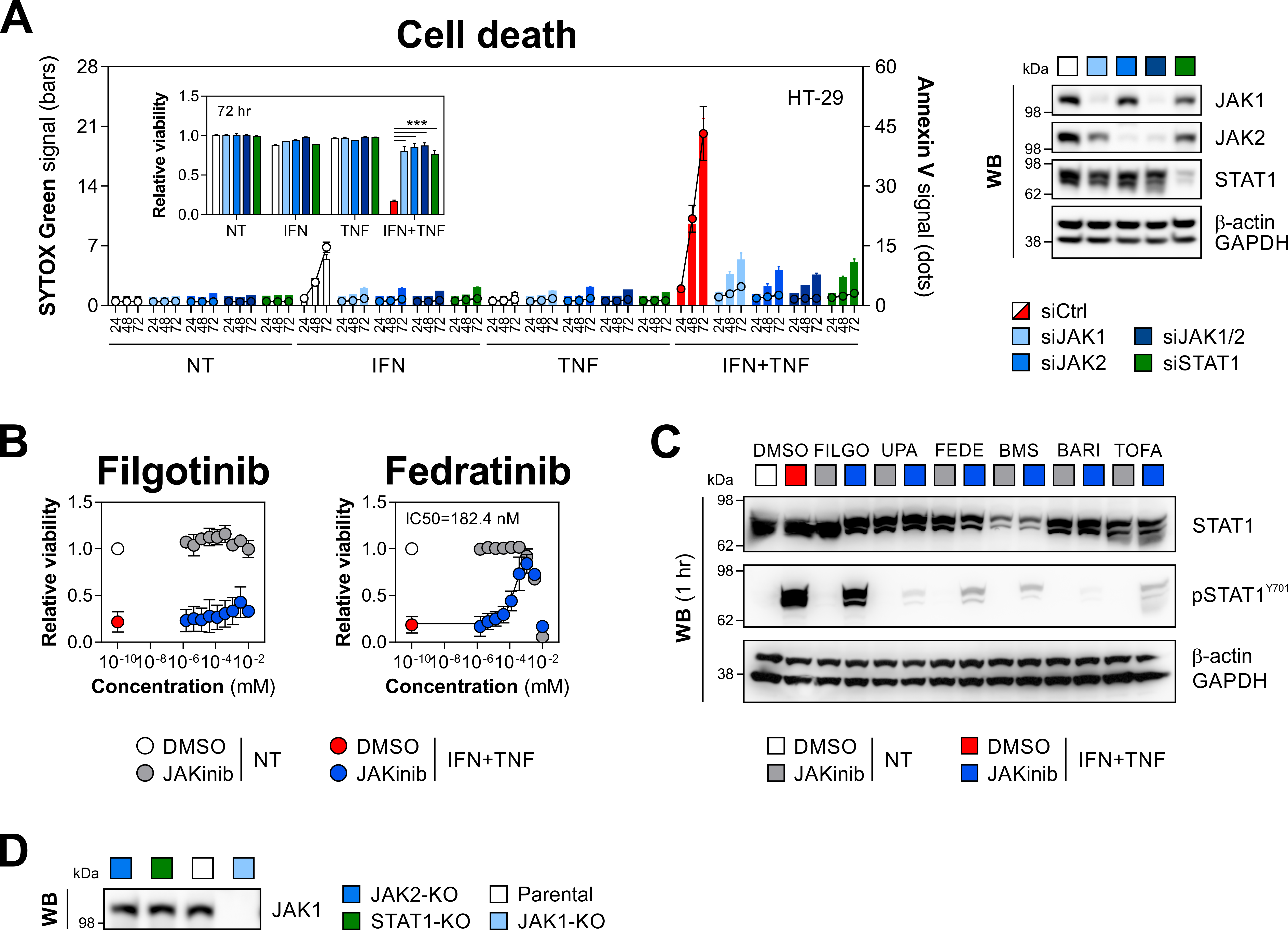

Supplement: Supplementary file 3 — Supplementary Figure S2 [file 41419_2021_4151_MOESM3_ESM.tif]

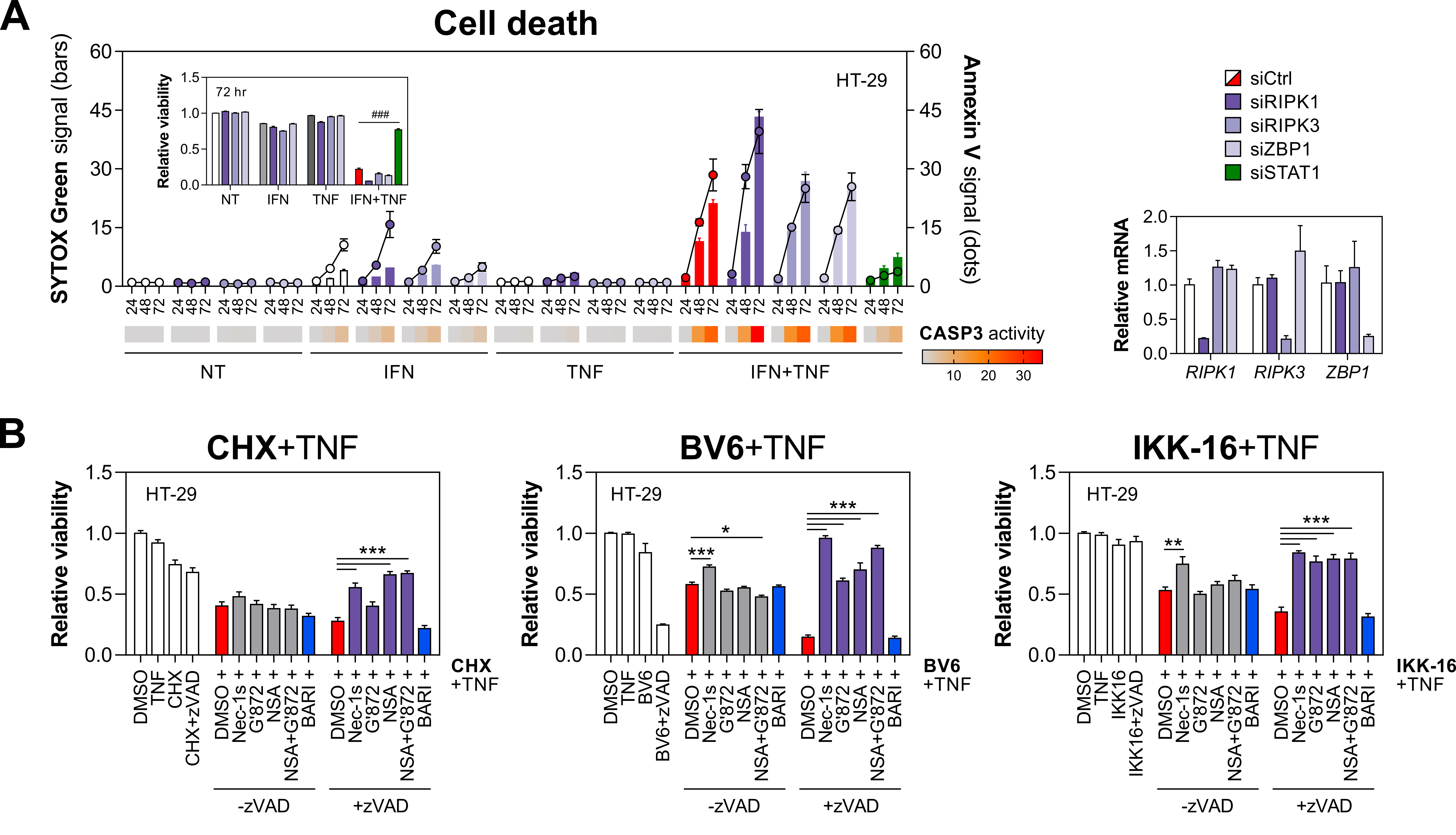

Supplement: Supplementary file 4 — Supplementary Figure S3 [file 41419_2021_4151_MOESM4_ESM.tif]

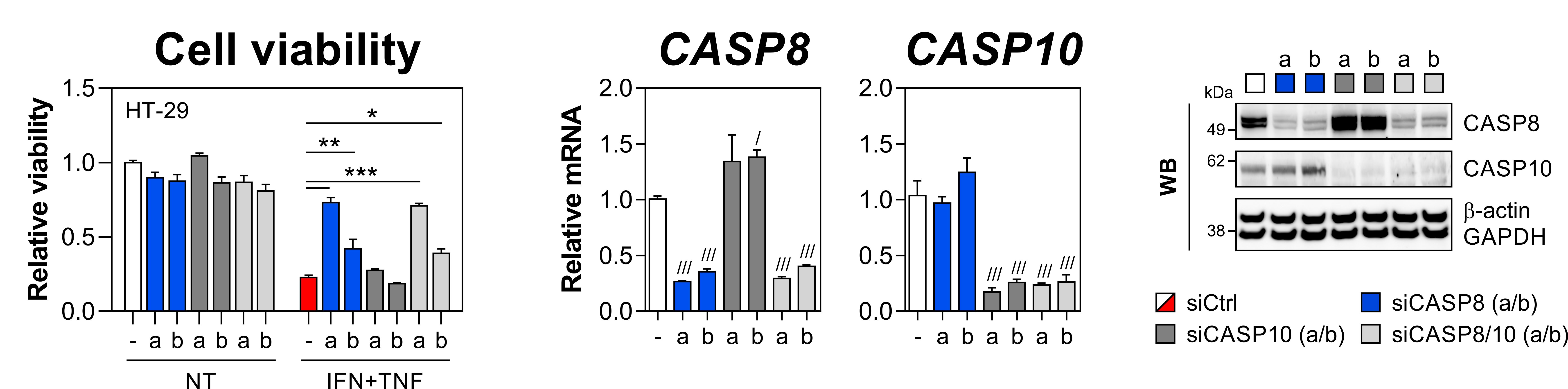

Supplement: Supplementary file 5 — Supplementary Figure S4 [file 41419_2021_4151_MOESM5_ESM.tif]
